# Supplementary material for: Restoration of Hepatic and Intestinal Integrity by Phyllanthus amarus Is Dependent on Bax/Caspase 3 Modulation in Intestinal Ischemia-/Reperfusion-Induced Injury
Source: Molecules. 2022 Aug 9;27(16):5073. doi: 10.3390/molecules27165073 (PMC9413108; doi:10.3390/molecules27165073)
Supplement: Supplementary file 1 [file molecules-27-05073-s001.zip › molecules-1720458-supplementary.pdf]

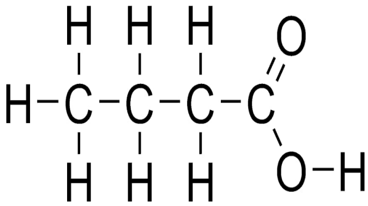

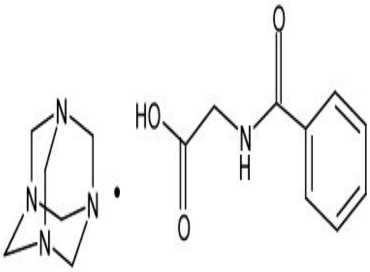

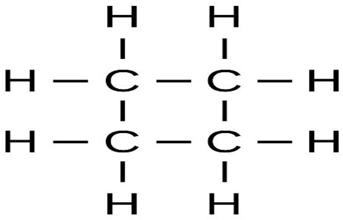

Butanoic **Methenamine** **Cyclobutane**

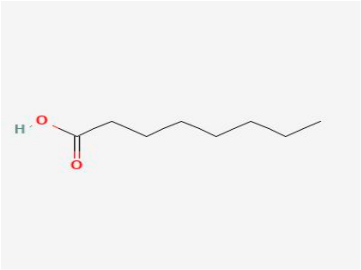

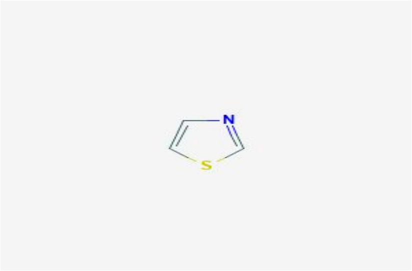

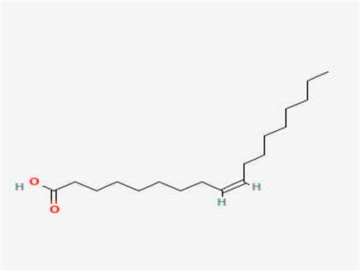

Octanoic Acid **Thiazole** **Oleic Acid**

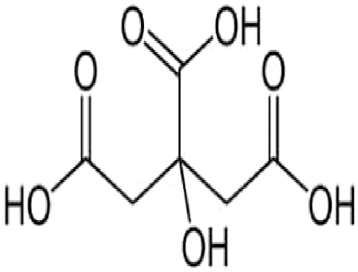

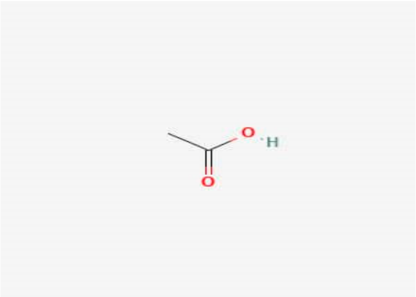

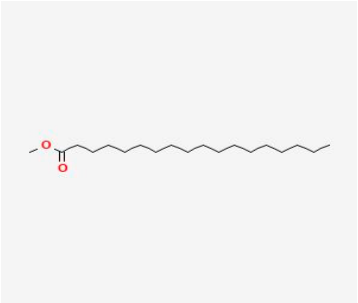

Citric Acid **Acetic Acid** **Hexadeconoic acid**

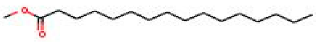

Methyl stearate

Supplementary Figure S1. Chemical structures of the Organic constituents of methanolic *P. Amarus* leaf extract

Supplementary Table S1. Intestinal histomorphometric analysis using Chiu's grading

Grade	Features
0	Normal mucosal villi.
1	Development of subepithelial Gruenhagen's space, usually at the apex of the villus; often with capillary congestion.
2	Extension of the subepithelial space with moderate lifting of epithelial layer from the lamina propria.
3	Massive epithelial lifting down the sides of villi. A few tips may be denuded.
4	Denuded villi with lamina propria and dilated capillaries exposed. Increased cellularity of lamina propria may be noted.
5	Digestion and disintegration of lamina propria; hemorrhage and ulcération.

Supplementary Table S2. Hepatic histomorphometric analysis using Eckhoff's grading

Grade	Features
**0**	Minimal or no evidence of injury
**1**	Mild injury consisting of cytoplasmic vacuolation and focal nuclear pyknosis
**2**	Moderate to severe injury with extensive nuclear pyknosis, cytoplasmic hypereosinophilia, loss of intercellular borders, and mild to moderate neutrophil infiltration
**3**	Severe injury with disintegration of hepatic cords, hemorrhage, and severe PMN infiltration
